# Robust skyrmion mediated reversal of ferromagnetic nanodots of 20 nm lateral dimension with high M_s_ and observable DMI

**DOI:** 10.1038/s41598-021-99780-1

**Published:** 2021-10-22

**Authors:** Md Mahadi Rajib, Walid Al Misba, Dhritiman Bhattacharya, Jayasimha Atulasimha

**Affiliations:** 1 Department of Mechanical and Nuclear Engineering, Virginia Commonwealth University, Richmond, VA 23284 USA; 2 Department of Electrical and Computer Engineering, Virginia Commonwealth University, Richmond, VA 23284 USA

**Keywords:** Magnetic devices, Magnetic properties and materials

## Abstract

Implementation of skyrmion based energy efficient and high-density data storage devices requires aggressive scaling of skyrmion size. Ferrimagnetic materials are considered to be a suitable platform for this purpose due to their low saturation magnetization (i.e. smaller stray field). However, this method of lowering the saturation magnetization and scaling the lateral size of skyrmions is only applicable where the skyrmions have a smaller lateral dimension compared to the hosting film. Here, we show by performing rigorous micromagnetic simulation that the size of skyrmions, which have lateral dimension comparable to their hosting nanodot can be scaled by increasing saturation magnetization. Also, when the lateral dimension of nanodot is reduced and thereby the skyrmion confined in it is downscaled, there remains a challenge in forming a stable skyrmion with experimentally observed Dzyaloshinskii–Moriya interaction (DMI) values since this interaction has to facilitate higher canting  per spin to complete a 360° rotation along the diameter. In our study, we found that skyrmions can be formed in 20 nm lateral dimension nanodots with high saturation magnetization (1.30–1.70 MA/m) and DMI values (~ 3 mJ/m^2^) that have been reported to date. This result could stimulate experiments on implementation of highly dense skyrmion devices. Additionally, using this, we show that voltage controlled magnetic anisotropy based switching mediated by an intermediate skyrmion state can be achieved in the soft layer of a ferromagnetic p-MTJ of lateral dimensions 20 nm with sub 1 fJ/bit energy in the presence of room temperature thermal noise with reasonable DMI ~ 3 mJ/m^2^.

## Introduction

Skyrmions are particle-like localized spin structures which can potentially overcome pinning with much smaller currents compared to domain walls (DW)^1–7^. This characteristic provides a pathway to implement energy efficient racetrack devices^7–10^. Skyrmions confined in the free layer of a Magnetic Tunnel Junction (MTJ) switched by electrical fields can also function as memory devices^11,12^. For example, skyrmions can assist voltage controlled magnetic anisotropy (VCMA) switching of perpendicular ferromagnets from up/down state to down/up state acting as an intermediate state^13–15^. This skyrmion mediated VCMA reversal has been shown to be robust while not requiring a bias magnetic field^13^. This could lead to scalable and energy efficient (< 1fJ/bit) VCMA switched MTJs ^13,15,16^. However, aggressive scaling of skyrmion size, both in racetrack and MTJ devices, is required to make them competitive with existing spin-transfer torque magnetic random-access memory (STT-MRAM) devices in terms of density and energy efficiency.

Multilayer thin ferromagnetic film stacks have been optimized to reduce skyrmion size down to ~ 30 nm starting from several micrometer lateral dimensions at room temperature^17–20^. Unfortunately, large stray fields originating from ferromagnets impede further scaling of skyrmions in thin films^21^. In confined structures, it has been previously shown that creation, annihilation and dynamics of skyrmions is drastically different from thin films due to the influence of geometric boundary on skyrmion stability and dynamics^6,22,23^. Though there have been few studies on skyrmions in a confined structure^24–27^, no prior work studied the requirement of both saturation magnetization (M_s_) and Dzyaloshinskii–Moriya Interaction (DMI) as the lateral dimension is downscaled to very small sizes ~ 20 nm. We previously showed ferromagnetic skyrmions cannot be formed in a 20 nm nanodot with experimentally observed DMI (~ 3.3 mJ/m^2^^28^) and require extremely large DMI (~ 13 mJ/m^2^)^15^. Hence, compensated ferrimagnets have recently emerged as an alternative to ferromagnets for hosting smaller skyrmions due to their small stray fields^29,30^. Skyrmion as small as ~ 10 nm has been observed in ferrimagnetic thin film of Co_56_Gd_44_ at room temperature^29^. However, in this work we show that high saturation magnetization favors formation of skyrmions in confined structures of small lateral dimensions (e.g. nanodots ~ 20 nm) as opposed to the case of ferrimagnetic thin films where weak stray fields due to low net saturation magnetization helps form smaller skyrmions. We also show that thermally robust skyrmion mediated switching can be attained in a 20 nm nanodot with high saturation magnetization and DMI values ~ 3 mJ/m^2^; both material parameters have been experimentally realized^28,31,32^.

## Results and discussion

### Formation of skyrmion in smaller nanodots requires higher M_s_ and DMI (simulations at 0 K)

**Figure 1:**
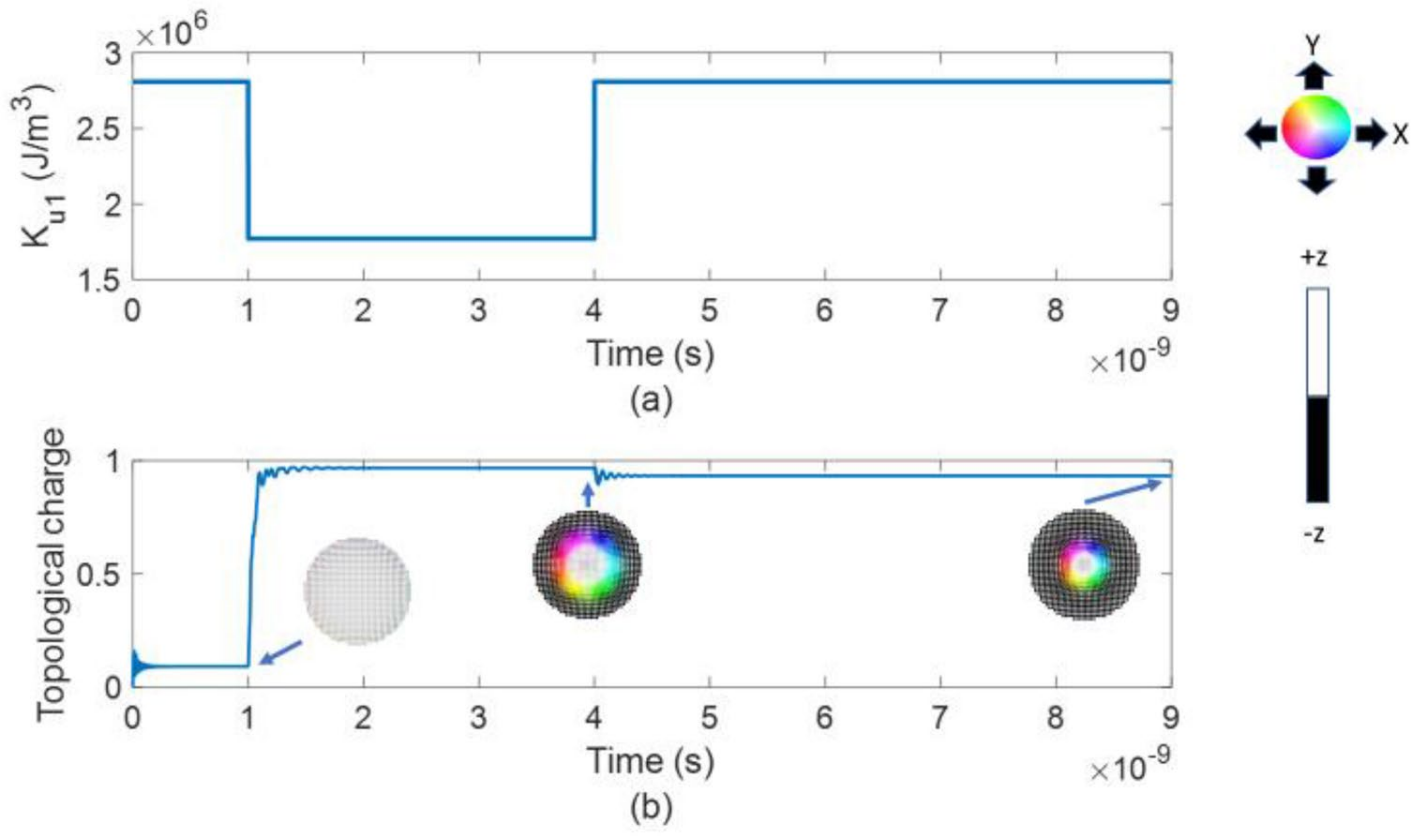
(**a**) Application of VCMA, (**b**) A quasi-ferromagnetic (QFM) state is created after relaxing for 1 ns. After the reduction of PMA, a skyrmion is formed and it is stable even after the withdrawal of voltage pulse.

To study the formation of a stable skyrmion we simulated the magnetization dynamics of circular nanodots of 50 nm, 30 nm and 20 nm diameter in response to an applied voltage pulse as shown in Fig. 1a using the micromagnetic simulation software Mumax3^33^. In all three cases, we initiate the simulations from a purely ferromagnetic state (all the spins pointing in the + z direction) and relax for 1 ns which ends up as a quasi-ferromagnet (QFM) state as shown in Fig. 1b. We can see that in a QFM state the boundary spins are slightly tilted. The perpendicular magnetic anisotropy (PMA) is then reduced through VCMA in 0.5 ps (see Supplementary Information section 1). After 3 ns, the voltage pulse is withdrawn and the PMA is restored to the initial value as shown in Fig. 1a. We see the equilibrium state after 5 ns and observe that the final state is either a QFM or a skyrmion. We find that after the withdrawal of voltage pulse, 5 ns is sufficient for reaching a stable state. This was confirmed by studying one equilibrium state (M_s_ = 1650 kA/m, DMI = 3.2 mJ/m^2^) for 1 µs and there was no change in average out-of-plane magnetization component or topological charge from those obtained after 5 ns. To find the required saturation magnetization (M_s_) and DMI as the lateral dimension of the nanodot is varied, the initial and reduced effective perpendicular anisotropy energy along with other parameters listed in Table 1 of the method section are kept constant. The effective PMA energy is expressed as $K_{eff} V = \left( {K_{u1} - \frac{1}{2}\mu_{0} M_{s}^{2} } \right)V$, where *K*_*eff*_ is the effective PMA energy density, *K*_*u1*_ is the PMA constant (uniaxial anisotropy) and *V* is the volume of the nanodot. The saturation magnetization is varied from 700 kA/m to 1700 kA/m for all nanodots.

The stable state turns out to be either a QFM or a skyrmion state. Figure 1 shows an example case of formation of a stable skyrmion in a 20 nm nanodot through VCMA where M_s_ = 1650 kA/m and DMI = 3.2 mJ/m^2^. The skyrmion is created when the effective PMA energy is reduced and remains stable even after the restoration of effective PMA energy to the initial value by withdrawing the voltage pulse.

**Figure 2:**
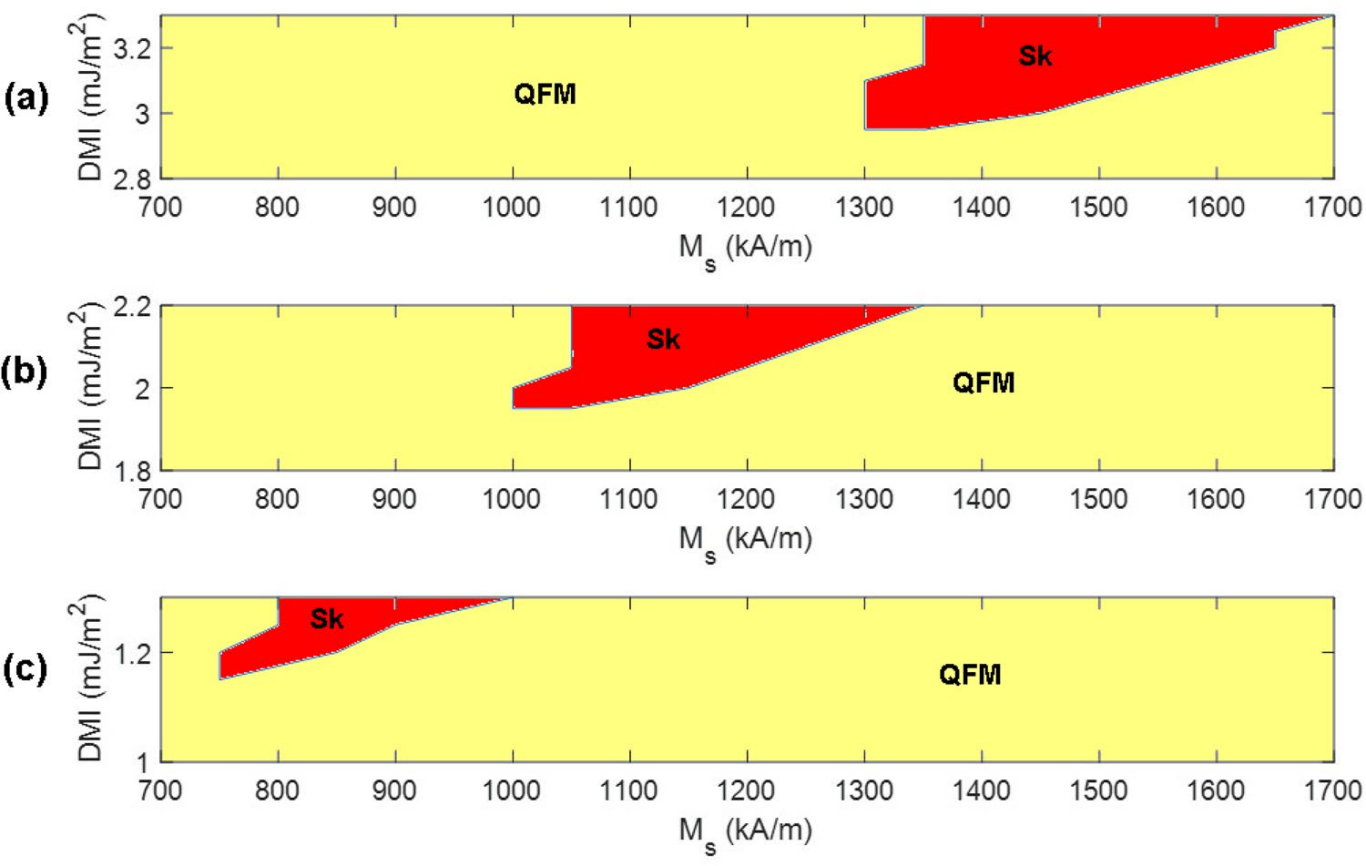
DMI-M_s_ phase diagram for (**a**) 20 nm, (**b**) 30 nm and (**c**) 50 nm (QFM and Sk in the phase diagrams represent the regions where quasi-ferromagnetic and skyrmion states are observed).

By following the strategy illustrated in Fig. 1a we observe the required M_s_ and DMI values for which skyrmions are formed in 20 nm, 30 nm and 50 nm nanodots. This is shown by DMI-M_s_ phase diagrams in Fig. 2.

It is understandable that in a 20 nm nanodot there will be higher tilting per spin to complete a 360° rotation along the diameter and the requirement of DMI will be higher compared to 30 nm and 50 nm nanodot for a constant effective PMA energy. Previously it has been shown that even for formation of transient skyrmion in a 20 nm nanodot, an extremely large DMI (~ 13 mJ/m^2^) is required^15^. So, it becomes a challenge to create a skyrmion in a 20 nm nanodot with experimentally observed DMI. Therefore, we focus on exploring the minimum DMI value with which a stable skyrmion can be formed under a constant effective PMA energy while the DMI is within the observable limit. We also observe the required saturation magnetization associated with the minimum DMI to explore which minimum DMI and M_s_ combination helps stabilize skyrmions in  the nanodots. In addition to that, we explore some other DMI values close to the minimum DMI and observe the corresponding M_s_ values for the formation of a skyrmion as shown in Fig. 2.

Figure 2a shows the DMI-M_s_ phase diagram for a 20 nm nanodot. We can see that minimum DMI for creating a stable skyrmion with a constant effective PMA energy as mentioned in Table 1 is 2.95 mJ/m^2^. Saturation magnetization required to form the skyrmion at this DMI value is 1300–1350 kA/m. Below 2.95 mJ/m^2^ DMI, no skyrmion is formed. As the DMI value is increased, the M_s_ range for which skyrmion can be created becomes wider on the higher end of M_s_ values. Considering a 1 nm thick MgO layer and an application of a voltage pulse of 2.0 V, the VCMA coefficient required to form these skyrmions in a 20 nm nanodot is 312 fJ/Vm^34^, see Supplementary Information section 2.

We next increased the lateral dimension to 30 nm and explored the minimum DMI required to form skyrmions in the 700–1700 kA/m M_s_ range at the effective PMA energy equal to that of 20 nm. From Fig. 2b we can see that the required minimum DMI is 1.95 mJ/m^2^ and the M_s_ is 1000–1050 kA/m for the formation of skyrmions in a 30 nm nanodot. At higher DMI, the M_s_ range for which the skyrmion is created grows wider on the higher end of M_s_ values similar to the 20 nm case.

The DMI-M_s_ relation required for formation of a stable skyrmion in increased lateral dimension nanodot of  50 nm is shown in Fig. 2c. Minimum DMI required for the formation of a stable skyrmion is 1.15 mJ/m^2^ and corresponding M_s_ value is 750 kA/m. Considering the MgO layer thickness and application of voltage pulse are the  same as 20 nm, the VCMA coefficients required for skyrmion formation in 30 nm and 50 nm nanodots are 139 fJ/Vm and 50fJ/Vm respectively.

For 50 nm, though a skyrmion is observed for a minimum DMI value of 1.15 mJ/m^2^ and M_s_ value of 750 kA/m,  no skyrmion is found if the DMI is kept constant at 1.15 mJ/m^2^ and M_s_ is increased. For other DMI values which are close to the minimum DMI, the required M_s_ remains in the comparatively lower end of M_s_ values. For 20 nm, the minimum DMI  occurs at high M_s_ values relative to the 30 nm and 50 nm lateral dimension cases.  For other DMI values close to the minimum DMI, the required M_s_ values are also on the higher end of M_s_ range. Expectedly, minimum DMI and M_s_ values for 30 nm fall in between those values required for 20 nm and 50 nm. Since the minimum DMI is associated with high M_s_ for 20 nm nanodots, we can say that large saturation magnetization helps stabilize skyrmions in smaller nanodots. This trend of downscaling of skyrmion lateral dimension draws a significant contrast with the downscaling of skyrmion size in thin films where lowering the saturation magnetization helps form smaller skyrmion. However, in nanodots, high saturation magnetization helps stabilize smaller skyrmions.

**Figure 3:**
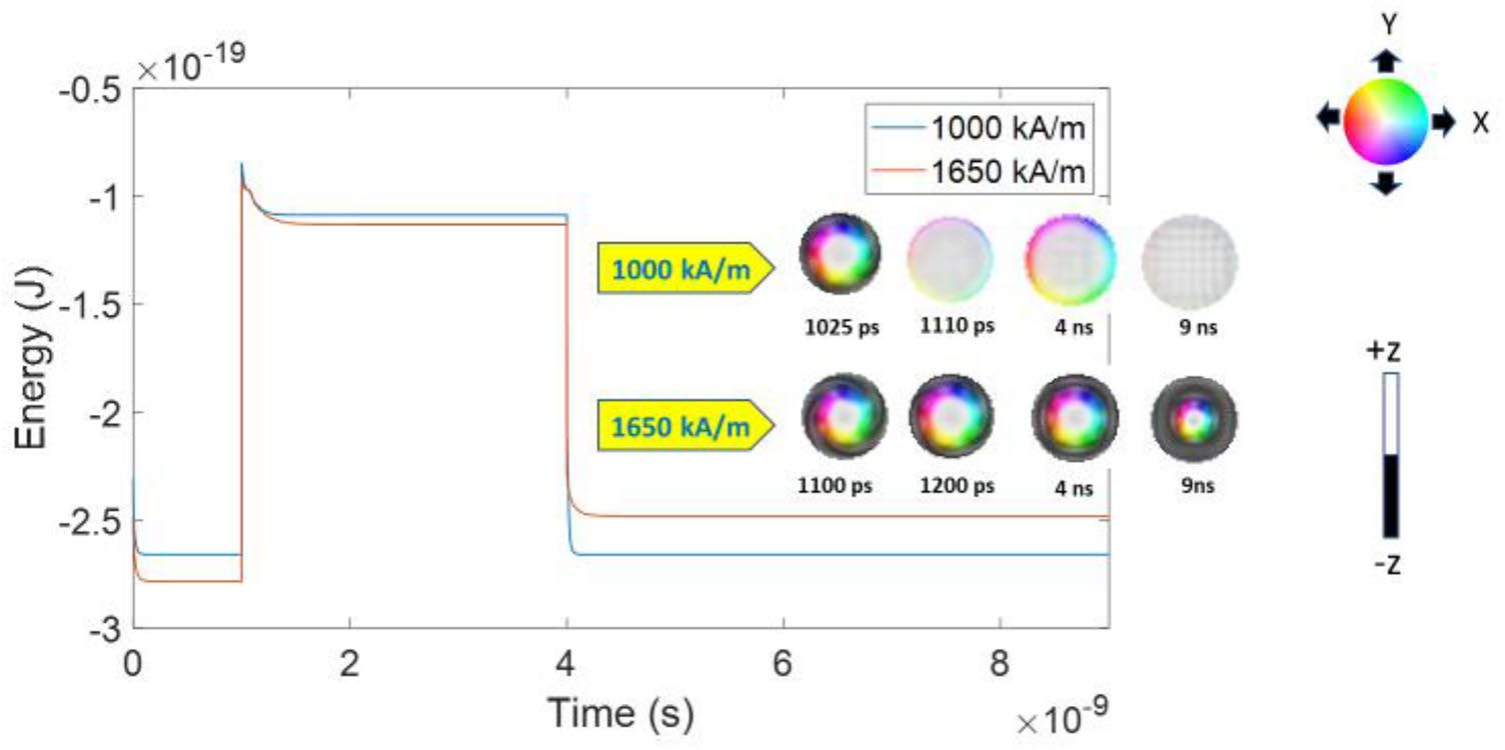
Evolution of total energy for two different M_s_ values and their corresponding magnetic states at different times.

To study how the skyrmions are formed at high M_s_ in downscaled nanodots, we observe the dynamic evolution and total energy of the corresponding states for 20 nm nanodot for various M_s_ values. We take two M_s_ values, one from the higher end (1650 kA/m) and the other from lower end (1000 kA/m) of M_s_ range at a constant DMI (3.2 mJ/m^2^) and observed the dynamic evolution of the magnetic states and their corresponding energy as shown in Fig. 3. For 1000 kA/m, skyrmion forms after 25 ps when PMA is reduced. The breathing of skyrmion is very fast and annihilates after ~ 100 ps at reduced PMA. After the annihilation of skyrmions, the nanodot stabilizes at QFM state. The stable QFM can be seen at 4 ns and this state has higher energy compared to the stable skyrmion formed with 1650 kA/m M_s_ at reduced PMA_._ When PMA is restored by withdrawing the voltage pulse, a stable ferromagnetic state is observed as shown at 9 ns in Fig. 3. For 1650 kA/m M_s_, a skyrmion forms slowly, specifically ~ 100 ps after the PMA is reduced. The skyrmion formed in this case also breathes slowly and stabilizes at this state.

**Figure 4:**
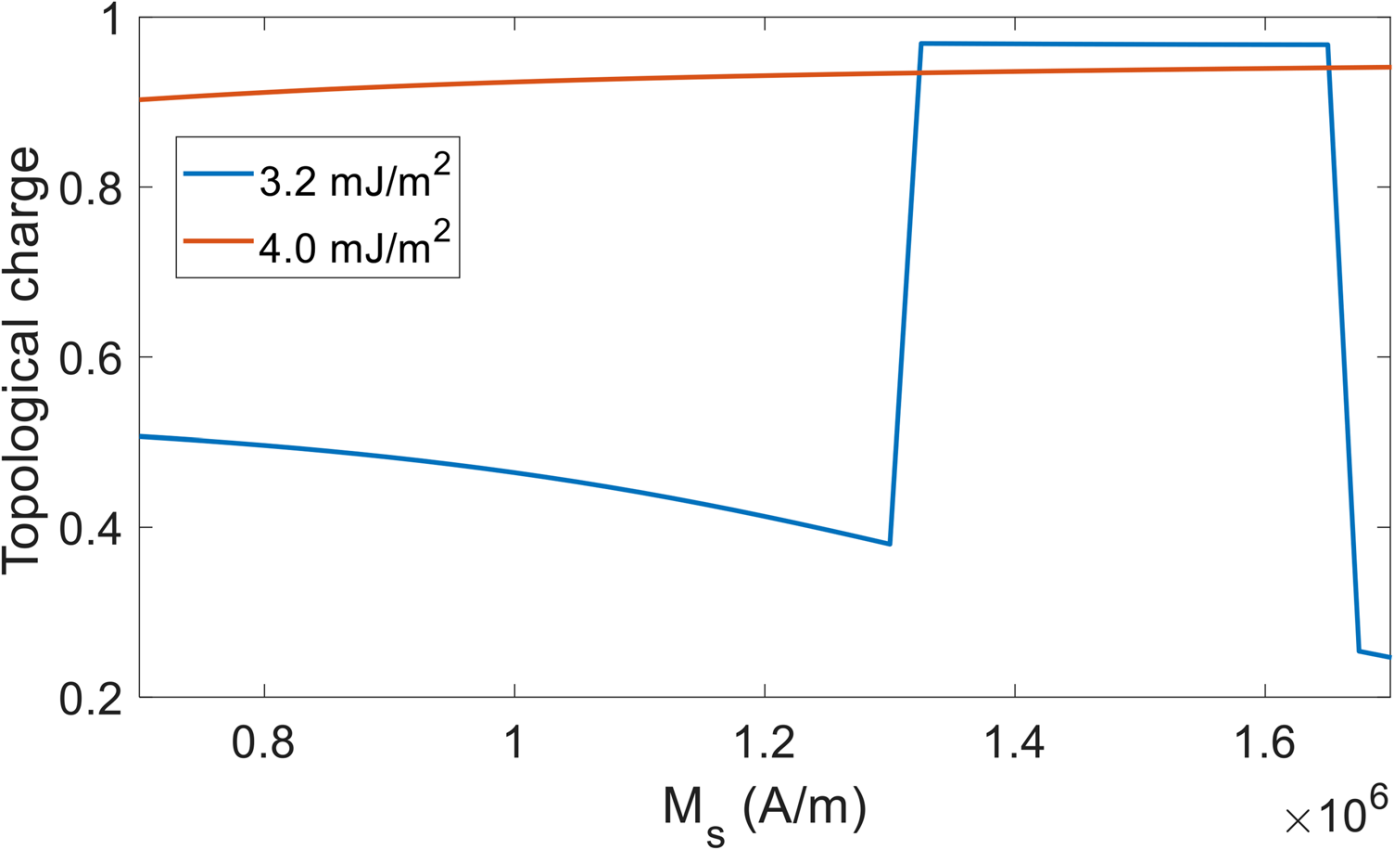
Topological charge vs. saturation magnetization of 20 nm nanodot for 3.2 mJ/m^2^ and 4.0 mJ/m^2^ DMI.

The requirement of higher DMI for decreased dimension of nanodots can be explained as follows. A higher DMI strength is required for completing the 360-degree rotation along a smaller diameter. Furthermore, when DMI is kept at the minimum required, high M_s_ is needed as it helps the formation of stray field dominated skyrmions in the confined nanostructure. Figure 4 shows that for 20 nm nanodots and 3.2 mJ/m^2^ DMI,  the minimum M_s_ at which a skyrmion is formed is 1350 kA/m whereas the  minimum M_s_ for the formation of skyrmion at 4.0 mJ/m^2^ DMI is only 700 kA/m. In summary, high M_s_ is not needed if we could employ high DMI and therefore form DMI mediated skyrmions. On the other hand, using low DMI requires a high M_s_ so that the stray field can aid the DMI in formation and stabilization of the skyrmions.

### Skyrmion-mediated voltage controlled switching of ~ 20 nm nanodot (with inclusion of room temperature thermal noise)

**Figure 5:**
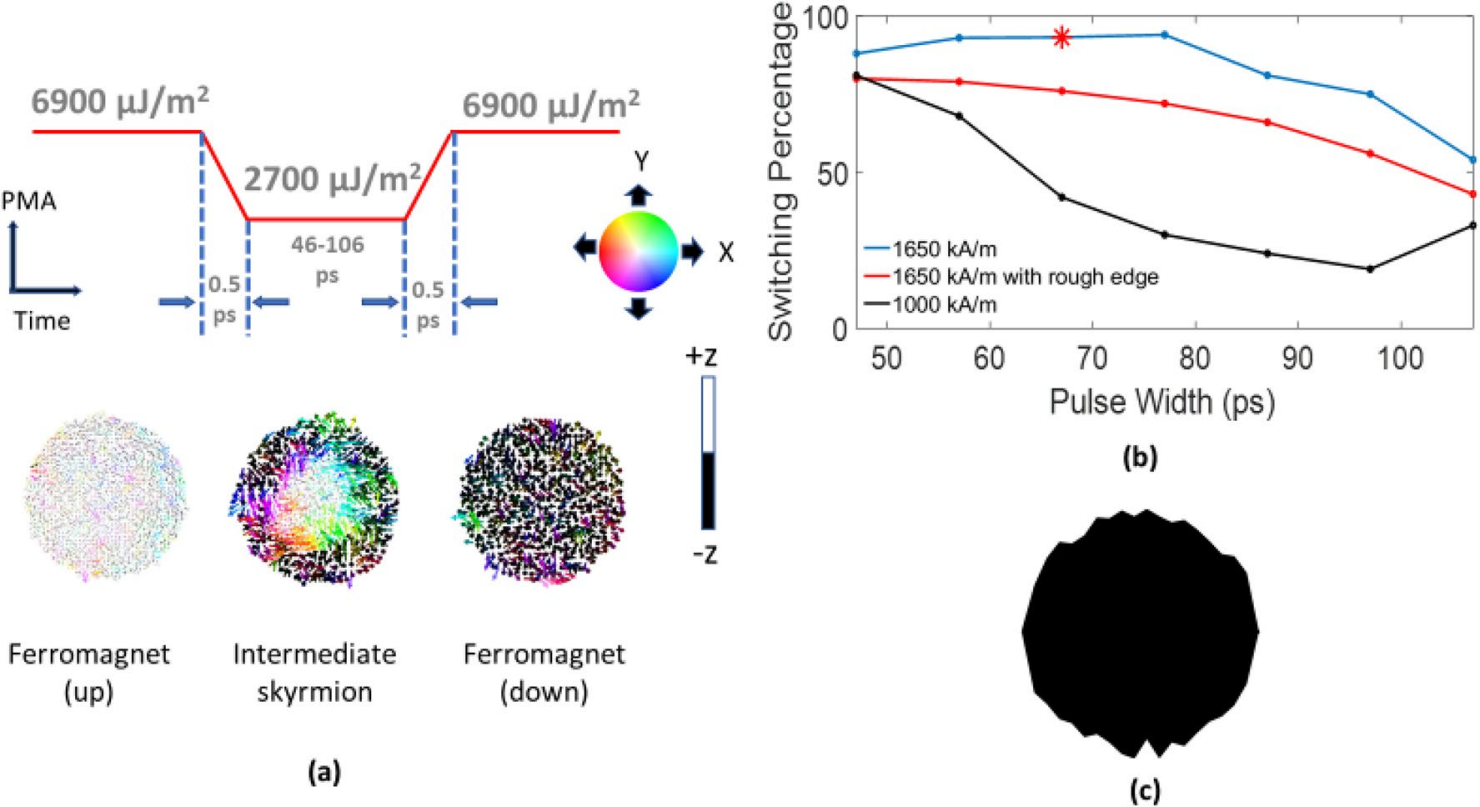
(**a**) Voltage pulse described in terms of PMA for switching of 20 nm nanodot in the presence of thermal perturbation; magnetization states corresponding to the PMA energy at different times are shown below the pulse diagram. (**b**) Switching percentage vs. pulse width (all of the points show the switching percentage for 100 simulation cases except the point marked with asterisk on the blue line. The star mark corresponds to a 67 ps pulse width where the simulations were run for 1000 times and 68 failures indicate ~ 93% switching) and (**c**) 20 nm nanodot with 5% edge roughness at the boundary.

In this section, we study the switching probability of 20 nm lateral dimension ferromagnetic nanodot by employing VCMA induced skyrmion mediated switching. In such switching, an intermediate skyrmion is created starting from a ferromagnetic state by lowering PMA, which is subsequently annihilated by restoring the PMA to achieve switching from ferromagnetic up/down to down/up state (Fig. 5a). This reversal mechanism is implemented in a similar way that is followed for stabilizing skyrmions but the only difference is in the timing of withdrawal of the voltage pulse. In stabilization, the voltage pulse is withdrawn after the skyrmion has been stabilized after some initial breathing at reduced PMA, whereas in reversal the voltage pulse is withdrawn while the skyrmion breathes. From stabilization in the absence of thermal noise, we have seen that for both cases of M_s,_ a skyrmion is formed. However, for lower value of M_s_ the breathing is very fast and the skyrmion thus formed cannot be stabilized. While skyrmion mediated magnetization reversal only requires a transient skyrmion, stabilization guides the choice of a skyrmion that breathes slowly, survives longer and provides potential robust switching.

For stabilization of skyrmions in the absence of thermal noise, the stability factor (*K*_*eff*_*V*/k_B_T) at reduced PMA is ~ 2.73. However, a larger stability factor is needed in the presence of thermal noise. So, the thickness of the 20 nm nanodot is increased from 0.6 to 1.5 nm to make the transient skyrmion thermally stable while keeping the reduced effective PMA energy density constant (60,000 J/m^3^) the same as non-thermal cases. Apart from increasing the thickness of the nanodot, the initial effective PMA energy density is also increased to ensure  a thermally stable ferromagnetic state. For studying the switching probability, we take two M_s_ values, 1000 kA/m and 1650 kA/m at a constant DMI 3.2 mJ/m^2^. The initial and reduced effective PMA energy are 330 k_B_T and 6.83 k_B_T respectively for the systems with M_s_ of 1000 kA/m and 1650 kA/m system. Therefore, the initial and reduced interfacial magnetic anisotropy (K_i_) for 1650 kA/m are 6900 μJ/m^2^ and 2700 μJ/m^2^ whereas the corresponding values for 1000 kA/m are 5300 μJ/m^2^ and 1000 μJ/m^2^ respectively. From non-thermal stabilization we observed that for both values of M_s_, initially a skyrmion is formed but for low value of M_s_ (1000 kA/m) this skyrmion does not stabilize. Since during  reversal a transient skyrmion suffices,  both low and high values of M_s_ are supposed to provide reversal if the voltage pulse is withdrawn synchronously  with the breathing of  the skyrmion. Therefore, we consider these two values of M_s_ to study which M_s_ value better favors the thermally robust switching . We let both systems relax for 100 ps to get the equilibrium state before trying to switch the magnetization. Due to the large PMA, this state is very close to the ferromagnetic state with very small canting of peripheral spins because of the stray field. After relaxing, we apply VCMA to reduce the PMA within 0.5 ps and a skyrmion is formed. We then restore the PMA by withdrawing the voltage pulse at different points (times in formation and dynamics of the skyrmion state) and observe switching. We note that Fig. 5a shows the switching from ferromagnet (up) to ferromagnet (down) state for M_s_ = 1650 kA/m and DMI = 3.2 mJ/m^2^ and the switching from ferromagnet (down) to ferromagnet (up) is shown in the Supplementary Information section 3. In this switching event, sub 1 fJ energy is dissipated on application of a voltage pulse of 2.0 V with 2130 fJ/Vm VCMA coefficient for a 1.5-nm-thick free layer and a 1-nm-thick MgO layer with relative permittivity ~ 7. We note that a high VCMA coefficient is needed as we chose a high initial PMA to ensure *K*_*eff*_*V*/k_B_T ~ 330. If we choose  a smaller *K*_*eff*_*V*/k_B_T ~ 68, which suffices for Random Access Memory (RAM), an interfacial PMA energy ~ 3500 μJ/m^2^ and VCMA coefficient ~ 405 fJ/Vm would be sufficient for the 1650 kA/m M_s_ system. We also note that for *K*_*eff*_*V*/k_B_T ~ 68, those required values would be well within the experimentally demonstrated values of interfacial PMA energy ~ 4060 μJ/m^2^^35^ and VCMA coefficient ~ 1043 fJ/Vm^36^. From Fig. 5b we can see that for a pulsewidth range of 47–107 ps, highest switching percentage for 1000 kA/m is 81% at 47 ps pulsewidth whereas for 1650 kA/m M_s_, ~ 93% switching can be achieved in 57–77 ps pulse width range. For both stabilization and thermal reversal of skyrmions, it appears that the boundary spins initiate the process of skyrmion formation whereas the edges of the circular nanodot are nearly smooth. So, we studied this system’s behavior in a nanodot where edge roughness is present. Therefore, we incorporated 5% edge roughness as shown in Fig. 5c at the boundary of the 20 nm nanodot to study the switching error in the same pulse width range of 47-107 ps. We note that the 5% edge roughness was created using a Gaussian distribution. From Fig. 5c we can see that in the presence of edge roughness for 1650 kA/m M_s_ and 3.2 mJ/m^2^ DMI, ~ 80% switching can be attained in the pulse width range of 47–57 ps. With better optimization of the material parameters (e.g. high M_s_ and experimentally observed DMI) and pulse shaping^37^, which is beyond the scope of this paper, higher switching percentage can be attained with sub fJ energy for each switching event. For example, the point in Fig. 5b with asterisk represents 93% switching at a pulse width of 67 ps.

In summary, higher DMI can result in formation of skyrmions in smaller nanodots at low M_s_ but such high values of DMI have not yet been experimentally observed. To create skyrmions in confined structures ~ 20 nm lateral dimensions with experimentally observed DMI ~ 3 mJ/m^2^, one needs large demagnetization (stray field) from materials that can be achieved with a high M_s_. We also showed that using a material with high saturation magnetization can help achieve thermally robust and extremely energy efficient switching in 20 nm ferromagnetic nanodot with experimentally demonstrated DMI. Thus, use of materials with high M_s_ can provide a pathway for aggressive scaling of ferromagnetic skyrmion mediated VCMA switching of p-MTJs with lateral dimensions ~ 20 nm and beyond.

## Method

The magnetization dynamics of circular nanodots was simulated by using the micromagnetic simulation software Mumax3^33^ for 50 nm, 30 nm and 20 nm lateral dimensions with a constant cell size of 0.5 × 0.5 × 0.6 nm^3^. In the Mumax3 framework, the magnetization dynamics is simulated by solving the Landau–Lifshitz–Gilbert (LLG) equation:

1 $$\frac{{\partial \vec{m}}}{\partial t} = \left( {\frac{ - \gamma }{{1 + \alpha^{2} }}} \right)\left[ {\vec{m} \times \vec{B}_{eff} + \alpha \left\{ {\vec{m} \times \left( {\vec{m} \times \vec{B}_{eff} } \right)} \right\}} \right]$$

where $\alpha$ is the Gilbert damping coefficient and $\gamma$ is the gyromagnetic ratio (rad/Ts). $\vec{m}$ is the normalized magnetization vector ($\vec{M}/M_{s}$), M_s_ is the saturation magnetization and $\vec{B}_{eff}$ is the effective magnetic field having the following components^33^:

2 $$\vec{B}_{eff} = \vec{B}_{demag} + \vec{B}_{exchange} + \vec{B}_{dm} + \vec{B}_{anis} + \vec{B}_{thermal}$$

Here, $\vec{B}_{demag}$ is the effective field due to demagnetization energy and $\vec{B}_{exchange}$ is the Heisenberg exchange interaction.

$\vec{B}_{dm}$ yields the Dzyaloshinskii–Moriya interaction:

3 $$\vec{B}_{dm} = \frac{2D}{{M_{s} }}\left( {\frac{{\partial m_{z} }}{\partial x},\frac{{\partial m_{z} }}{\partial y}, - \frac{{\partial m_{x} }}{\partial x} - \frac{{\partial m_{y} }}{\partial y}} \right)$$

where m_x_, m_y_ and m_z_ are the normalized magnetization components along the three cartesian co-ordinates and D represents the DMI constant (J/m^2^).

$\vec{B}_{anis}$, the effective field due to perpendicular anisotropy, is given by the following equation:

4 $$\vec{B}_{anis} = \frac{{2K_{u1} }}{{M_{s} }}\left( {\vec{u}.\vec{m}} \right)\vec{u}$$

Here $K_{u1}$ indicates first order uniaxial anisotropy constant and $\vec{u}$ stands for a unit vector in the anisotropy direction. The temperature effect is calculated by:

5 $$\vec{B}_{thermal} = \vec{\eta }\left( {step} \right)\sqrt {\frac{{2\alpha k_{B} T}}{{M_{s} \gamma \Delta V\Delta t}}}$$

where T is the temperature (K), $\Delta V$ is the cell volume, *k*_*B*_ is the Boltzmann constant, $\Delta t$ is time step and $\vec{\eta }\left( {step} \right)$ is a random vector from a standard normal distribution which is independent (uncorrelated) for each of the three cartesian co-ordinates. Its value is changed after every time step.

The parameters listed in Table 1 were used for all of the simulation cases unless otherwise stated.

**Table 1:** List of parameters.

Exchange stiffness	5 pJ/m^38^
Thickness	0.6 nm
Gilbert damping coefficient	0.05^39^
Cell size	0.5 nm × 0.5 nm × 0.6 nm
Initial effective PMA energy	1.30 eV
Reduced effective PMA energy	0.07 eV

## Supplementary Information

Supplementary Information.
