# Supplementary material for: Robust skyrmion mediated reversal of ferromagnetic nanodots of 20 nm lateral dimension with high Ms and observable DMI
Source: Sci Rep. 2021 Oct 22;11:20914. doi: 10.1038/s41598-021-99780-1 (PMC8536757; doi:10.1038/s41598-021-99780-1)
Supplement: Supplementary file 1 — Supplementary Information. [file 41598_2021_99780_MOESM1_ESM.docx]

**Robust skyrmion mediated reversal of ferromagnetic nanodots of 20 nm lateral dimension with high M_s_ and observable DMI**

Md Mahadi Rajib^1^, Walid Al Misba^1^, Dhritiman Bhattacharya^1^ and Jayasimha Atulasimha^1,2^

*^1^Department of Mechanical and Nuclear Engineering, Virginia Commonwealth University, Richmond, VA 23284, USA*

*^2^Department of Electrical and Computer Engineering, Virginia Commonwealth University, Richmond, VA 23284, USA*

* Corresponding author: [jatulasimha@vcu.edu](mailto:jatulasimha@vcu.edu)

**Supplementary material for Robust skyrmion mediated reversal of ferromagnetic nanodots of 20 nm lateral dimension with high M_s_ and observable DMI**

**S1 Feasibility of achievement of 0.5 ps ramp time**

In the main paper, we have stated that PMA is reduced through VCMA in 0.5 ps. Achievement of such small ramp time requires a small RC time constant, which in turn depends on the availability of small resistance area (RA) product. Considering 1nm thick MgO layer, the required RA product (~8 Ω.µm^2^) for achieving 0.5 ps ramp time is within experimentally reported values [1, 2].

**S2 Calculation of VCMA coefficient**

VCMA coefficient, η, can be calculated from [3]:

$$\eta=\frac{\triangle PMA\times t_{free}}{\frac{\triangle V}{t_{MgO}}}$$

Where △PMA, *t_free_*, △V and *t_MgO_* represent modulation of perpendicular magnetic anisotropy, thickness of the free layer, applied voltage pulse and thickness of the MgO layer respectively. For an example, considering a voltage pulse of 2V and 1 nm thick MgO layer, the VCMA coefficient required for 20nm nanodot is, η= 312 fJ/Vm. We note that thickness of the free layer (0.6 nm) along with initial and reduced effective PMA energy are listed in Table 1 from where the PMA modulation can be obtained by dividing the difference of these two energies with the volume of the nanodot.

**S3 Switching from ferromagnet (down) to ferromagnet (up)**

In Fig. 5a of main manuscript switching is shown from ferromagnet (up) to ferromagnet (down) state. In this switching event the intermediate skyrmion has polarity, p=-1. Supplementary Figure 1 shows the switching from ferromagnet (down) to ferromagnet (up) state where the intermediate skyrmion has polarity, p= 1.

**
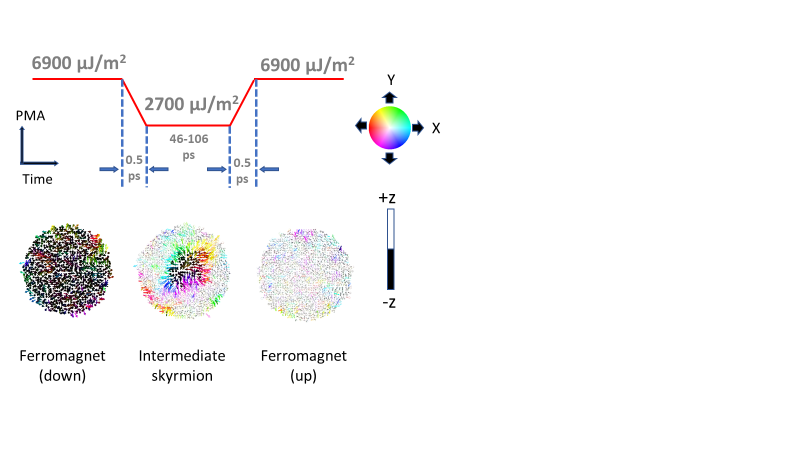
**

**Supplementary Figure 1. Switching from ferromagnet (down) to ferromagnet (up)**

Voltage pulse in terms of PMA and switching of 20 nm nanodot in the presence of thermal perturbation from ferromagnet (down) to ferromagnet (up); magnetization states corresponding to the PMA energy at different time are shown below the pulse diagram.

**References:**

1. Jan, G., Thomas, L., Le, S., Lee, Y.-J., Liu, H. et al. Demonstration of ultra-low voltage and ultra low power STT-MRAM designed for compatibility with 0x node embedded LLC applications. *IEEE Symposium on VLSI Technology (2018).* DOI: <https://doi.org/10.1109/VLSIT.2018.8510672>
2. Park, C., Lee, H., Ching, C., Ahn, J., Wang, R., Pakala, M. & Kang, S. H. Low RA magnetic tunnel junction arrays in conjunction with low switching current and high breakdown voltage for STT-MRAM at 10 nm and beyond. *IEEE Symposium on VLSI Technology* (2018). DOI: https://doi.org/10.1109/VLSIT.2018.8510653.
3. Bhattacharya, D. & Atulasimha, J. Skyrmion-mediated voltage-controlled switching of ferromagnets for reliable and energy-efficient two-terminal memory. *ACS Appl. Mater. Interface* **10,** no. 20, 17455-17462 (2018).
